# Quality Assessment of Web-Based Information Related to Diet During Pregnancy in Pregnant Women: Cross-Sectional Descriptive Study

**DOI:** 10.2196/64630

**Published:** 2025-06-03

**Authors:** Daichi Suzuki, Etsuko Nishimura, Rina Shoki, Ishak Halim Octawijaya, Erika Ota

**Affiliations:** 1 Global Health Nursing Graduate School of Nursing Science St. Luke's International University Tokyo Japan; 2 Institute for Global Health Policy Research (iGHP) Bureau of Global Health Cooperation Japan Institute for Health Security Tokyo Japan; 3 Department of Nursing Faculty of Nursing Komazawa Women's University Tokyo Japan; 4 Department of Nursing Faculty of Nursing at Saitama Japanese Red Cross College of Nursing Saitama Japan; 5 School of Nutrition and Dietetics Faculty of Health and Social Services Kanagawa University of Human Services Kanagawa Japan

**Keywords:** assessment, availability, decision-making, diet, dietary information, internet, internet-based, misinformation, nutrition, nutrition-related, online health information, physical harm, pregnancy, pregnancy-related guidance, pregnant women, prenatal nutrition, psychological harm, QUEST, quality assessment, tools, web-based, web-based information, website assessment, women’s health

## Abstract

**Background:**

The widespread availability of health information online, coupled with the ease of access to the internet, has led pregnant women to rely heavily on online sources for pregnancy-related guidance. The internet-based information regarding nutrition enabled positive dietary changes for pregnant women. Although there are some important sources for pregnant women to collect their health information, some information increases maternal anxiety and difficulties based on a lack of information. Moreover, some women become confused due to conflicts on the same topics from different websites. However, concerns about the reliability and impact of this information have surfaced, contributing to heightened anxiety among expectant mothers. The importance of the quality of web-based information is increasingly recognized; however, no studies have evaluated the quality of nutrition-related information for pregnant women.

**Objective:**

This study aims to bridge this research gap by assessing the quality of online health information concerning prenatal nutrition tailored to pregnant women.

**Methods:**

This cross-sectional descriptive study was conducted through a Google keyword search on February 14, 2023. We used search terms, such as “pregnancy,” “pregnant women,” “diet,” and “nutrition” and conducted an exhaustive search on Google. Using the Quality Evaluation Scoring Tool (QUEST), we meticulously evaluated the quality of the retrieved information.

**Results:**

The top 20 Google-searched sites were evaluated using the QUEST tool. The average score was 11.7 points, ranging from 6 to 15, with most sites scoring between 11 and 15. Half of the websites lacked clear authorship and most gave weak or no attribution to specific scientific sources. While conflict of interest scored highest overall, with 60% showing no bias, some sites promoted products or specific interventions. Currency was inconsistent—only half were updated within 5 years. Complementarity received the lowest scores, with 70% lacking support for patient-physician relationships. The tone was generally positive, with 95% supporting their claims, though only one site used a balanced, well-reasoned tone. Discrepancies in cited guidelines on nutritional intake and inappropriate expressions about alcohol, weight management, and miscarriage raised concerns about the information’s accuracy and appropriateness.

**Conclusions:**

Although many websites use cautious language to mitigate commercial influence, deficiencies persist in crucial areas for empowering informed decision-making among pregnant women. From our assessment of the results, it was found that incorrect evidence information is provided at the top of search results, which is easily accessible to users. The inadequacies in attributing authorship, clarifying conflicts of interest, and ensuring the currency of information pose substantial challenges to the reliability and usefulness of online health resources in prenatal nutrition. Since internet-based information is the most accessible, reliable evidence should be provided to protect everyone from misinformation, including shallow health literacy demographics, and from potential physical and psychological harm.

## Introduction

Internet penetration rates are high in developed countries, resulting in easy web access for a substantial portion of the population, including pregnant women. In Japan, the internet usage rate reached 82.9% in 2021, with smartphones being the most prevalent devices (68.5%), followed by computers (48.1%) [1]. Owing to its accessibility, many individuals at different life stages, including pregnant women, depend on the internet to obtain health-related information. Among women receiving antenatal care in a hospital in Ireland, only 3% did not own a smartphone, but all of them had internet access to seek the information they needed [2].

Most women used the internet to access pregnancy-related information and found it beneficial [3]. Approximately 96% of pregnant women in Canada use the internet to find information about nutrition during pregnancy, with 75% reporting that they use the internet more often than any other source [4]. Women who are in their mid-20s to 30s, who were educated after high school, are employed, and are first-time mothers are more likely to obtain nutrition-related information from the internet [5]. Another study found that women with relatively low educational levels used websites less frequently than those with moderate or high levels of education. In addition, first-time mothers who reported higher levels of social support evaluated website information’s quality more favorably [6]. Pregnant women search various nutrition-related topics online, including food safety and healthy eating (nutrient intake and requirements, serving sizes, recipes and meal plans, foods to avoid, etc) [4,7].

Nutrition-related information is crucial for pregnant women to address significant physiological, psychological, and social changes during pregnancy [8]. Without the appropriate information, pregnant women, especially first-time mothers, may not have a healthy pregnancy. Moreover, among healthy eating habits, adequate nutrient intake is necessary to prevent prenatal weight gain problems for both the mother and baby and to avoid particular nutrient deficiencies [9]. First-time mothers in Australia are reported to consume inadequate fruits and vegetables but excess soft drinks and fast food, which puts them at risk of micronutrient deficiency. Such poor eating habits are especially observed among pregnant women with lower socioeconomic status [10]. Since pregnant women with socioeconomically disadvantaged backgrounds likely have poorer access to health care services, including nutrition counseling with registered dietitians or other medical professionals, internet-based nutrition information is important to trigger healthy eating habits, especially among first-time mothers. Thus, the internet serves as a key source of prenatal and maternal information regarding nutrition for all pregnant women, enabling them to make positive dietary changes based on the information they find online.

However, accessibility and convenience of internet-based nutritional information may face some barriers, such as increased anxiety regarding the content described, lack of trust, and difficulty in finding information. Some pregnant women worry about their eating habits after obtaining information from the internet (are they capable of providing the adequate nutrients necessary for their babies?) or become concerned when they find they have consumed something bad for their babies. In addition, some pregnant women have found contradictory nutrition-related information on different websites, which confuses them regarding the information’s reliability. Other women found it difficult to obtain accurate information on a specific topic. Improved access to trustworthy online sources and increased availability of information on different diets and health conditions can benefit pregnant women seeking nutrition-related information online [4].

A review of 18 Australian websites indicated that none were completely aligned with available evidence-based guidelines [11]. Although various online information exists on nutrition during pregnancy, no studies have evaluated the quality of this information in Japan. Therefore, this study aims to assess the quality of online health information on nutrition for pregnant women.

## Methods

### Selection of Websites

This cross-sectional descriptive study was conducted through a Google keyword search on February 14, 2023. Google Search is the most widely used search engine worldwide [12] and studies have used it to conduct research on health-related issues [13,14]. To search the websites, this study used terms such as “pregnancy,” “pregnant women,” “diet,” and “nutrition.” Based on these searches, the top 20 Japanese sites were selected for quality evaluation. Studies show that most internet users review only the top 10 search results [15]. To capture broader content, we analyzed the top 20 websites likely accessed for pregnancy-related nutrition information. Searches were conducted in incognito mode with cookies cleared to minimize personalization effects. Although the search was conducted in incognito mode with cookies cleared to minimize personalization, we acknowledge that this approach does not fully eliminate all forms of personalization. Factors such as IP address tracking, device fingerprinting, and browser configurations may still influence search results. In addition, Google’s search algorithm itself is not neutral and may introduce algorithmic biases that affect which websites appear at the top of search results. These factors could influence the representativeness and neutrality of the data collected [16,17].

### Quality Evaluations

The Quality Evaluation Scoring Tool (QUEST) is a reliable and valid tool for evaluating online articles on health [16]. The QUEST assessed the information quality of the websites based on 7 weighted criteria: authorship, attribution, study type, conflicts of interest, currency, complementarity, and tone. The QUEST emphasizes balanced evaluation, prioritizing key indicators such as attribution, conflict-of-interest disclosure, and tone [16]. Textbox 1 presents the details of each QUEST component. Higher scores indicate better information quality, with the maximum attainable score of 28 [16].

**Details of each component of the Quality Evaluation Scoring Tool.**

**Authorship (score × 1)**

0: No indication of authorship or username

1: All other indications of authorship

2: Author’s name and qualification clearly stated

**Attribution (score × 3)**

0: No sources

1: Mention of expert source, research findings (though with insufficient information to identify the specific studies), links to various sites, advocacy bodies, or other

2: Reference to at least 1 identifiable scientific study, regardless of format (eg, information in text and reference list)

3: Reference to mainly identifiable scientific studies, regardless of format (in >50% of claims)

For all articles scoring 2 or 3 on Attribution (Score x 1)

**Type of study**

0: In vitro, animal models, or editorials

1: All observational work

2: Meta-analyses, randomized controlled trials, or clinical studies

**Conflict of interest (score × 3)**

0: Endorsement or promotion of intervention designed to prevent or treat condition (eg, supplements, brain training games, and foods) within the article

1: Endorsement or promotion of educational products and services (eg, books and home care services)

2: Unbiased information

**Currency (score × 1)**

0: No date present

1: Article is dated but 5 years or older

2: Article is dated within the last 5 years

**Complementarity (score × 1)**

0: No support of the patient-physician relationship

1: Support of the patient-physician relationship

**Tone (includes title; score × 3)**

0: Fully supported (authors fully and unequivocally support the claims; use strong vocabulary such as “cure,” “guarantee,” and “easy”; mostly use nonconditional verb tenses such as “can” and “will”; and include no discussion of limitations)

1: Mainly supported (authors mainly support their claims but with more cautious vocabulary such as “can reduce your risk” or “may help prevent” and include no discussion of limitations)

2: Balanced/cautious support (authors’ claims are balanced by caution and include statements of limitations and/or contrasting findings)

In addition, content analysis was conducted on information regarding the recommended nutrient intake based on the “Dietary Reference Intakes for Japanese” (2020) [17] to examine each website’s information content and expressions. A total of 2 reviewers (EN and RS) rigorously reviewed the top 20 eligible websites using a predefined content analysis framework in Microsoft Excel to ensure consistent evaluation of “wording of concern” and “accuracy of information.” A health care professional with expertise in pregnancy and nutrition validated the analysis through repeated reviews. Prior to the full analysis, both reviewers conducted a pilot assessment on a subset of 5 websites to refine the coding framework and confirm a shared understanding of the evaluation criteria. Any discrepancies during the content analysis were resolved through consensus discussion, with additional input from a health care professional when necessary. This process ensured interrater agreement and strengthened the reliability of the evaluation.

### Conceptual Model

Developed for higher education students, the conceptual model of engagement in online learning [18] incorporates diverse perspectives and a multidimensional structure. The model comprises 5 engagement types: cognitive engagement, behavioral engagement, collaborative engagement, emotional engagement, and social engagement.

In the context of this research, pregnant women who seek health information online are engaging in what can be referred to as “individual health engagement,” which may be interpreted through the 5 elements of the aforementioned conceptual model.

The application of this model to pregnant women’s engagement types above and justified as follows:

1. Cognitive engagement: pregnant women critically assess and integrate the health information they encounter online.
2. Behavioral engagement: actively searching for online health information reflects the development of self-efficacy and skills in managing pregnancy.
3. Collaborative engagement: pregnant women may interact with peers or healthcare professionals through online platforms.
4. Emotional engagement: the act of seeking health information online can elicit a range of emotional responses related to pregnancy.
5. Social engagement: the pursuit of health information online can help foster a sense of community among pregnant women.
6. Collectively, this framework highlights the multifaceted nature of pregnant women’s engagement with online health information during pregnancy.

### Ethical Considerations

The data used are publicly available and provided anonymously. In addition, this study did not collect data on or include any human subjects. Therefore, it was deemed that ethical approval was not required. The content was public and excluded sensitive or proprietary information. The analysis focused on aggregated and anonymized evaluations, ensuring compliance with copyright laws.

## Results

### QUEST Assessment

Google Search yielded approximately 11,300,000 sites and the top 21 sites comprised 20 regular pages and one listed page, with no video content included. Therefore, we conducted a quality assessment of the 20 regular pages. Table 1 presents the QUEST scores for the 20 pages. The average score was 11.7, ranging from 6 to 15, with most sites scoring between 11 and 15. No significant outliers were identified. The criterion of complementarity had the lowest average score, with 70% of the pages scoring low in this attribute, followed by attribution, and authorship. In contrast, the average scores for conflict of interest, currency, and tone of the content were higher.

Regarding authorship, half of the pages had no information on authorship or usernames. However, the remaining pages clearly stated their authors’ information. In terms of attribution, only 1 page referred to at least one piece of scientific information. However, the remaining 65% (13 sites) referred to experts’ opinions, research papers, or other sources without any evidence to identify specific studies. Moreover, 30% (6 sites) of the sites provided no sources. Although 1 site cited some sources, it referred to editorials with no evidence. To examine conflict of interest, this category was reported to have the highest average score among all the criteria. Of the sites, 60% contained information with no biases. A total of 3 sites presented recommendations, such as on educational products or services, with some biases. Moreover, 25% (5 sites) recommended specific interventions. As for currency, half of the sites (10/20 sites) provided the date and the information was written within the past 5 years. In addition, while 1 site provided the date, the information was older than 5 years. Meanwhile, the remaining sites provided no data sources. On the subject of complementarity, the sites scored lowest on this criterion. That is, 70% of the sites scored low on this criterion, meaning no support was provided for patient-physician relationships.

Finally, regarding tone, 95% of the sites (19/20) supported their claims, but 1 page provided more support with balance and caution. The highest tone score was achieved by a municipal website that used balanced language and provided clear reasoning for its recommendations. Despite the higher average score for this criterion, 45% of the sites showed no date present in the currency assessment.

**Table 1:** Assessments of quality of information in all included webpages.

Authors’ assessment									Weighted score with authors’ assessment								Total score (up to 28)
Number	Authorship	Attribution	Type of study	Conflict of interest	Currency	Complementarity	Tone (includes title)	Authorship (score × 1)		Attribution (score × 3)	Type of study (score × 1)	Conflict of interest (score × 3)	Currency (score × 1)	Complementarity (score × 1)	Tone (includes title)		
1	0	1	–^a^	2	2	0	1	0		3	0	6	2	0	3	14	
2	2	０	–	2	0	0	1	2		0	0	6	0	0	3	11	
3	2	2	0	0	0	1	1	2		6	0	0	0	1	3	12	
4	2	0	–	0	0	1	1	2		0	0	0	0	1	3	6	
5	2	0	–	2	0	0	1	2		0	0	6	0	0	3	11	
6	2	1	–	1	2	0	1	2		3	0	3	2	0	3	13	
7	0	1	–	2	2	0	1	0		3	0	6	2	0	3	14	
8	0	1	–	2	0	0	1	0		3	0	6	0	0	3	12	
9	2	0	–	2	2	0	1	2		0	0	6	2	0	3	13	
10	2	1	–	1	2	0	1	2		3	0	3	2	0	3	13	
11	2	1	–	2	0	0	1	2		3	0	6	0	0	3	14	
12	0	0	–	0	2	1	1	0		0	0	0	2	1	3	6	
13	0	0	–	2	2	0	2	0		0	0	6	2	0	6	14	
14	2	1	–	0	0	0	1	2		3	0	0	0	0	3	8	
15	0	1	–	2	0	0	1	0		3	0	6	0	0	3	12	
16	0	1	–	2	1	0	1	0		3	0	6	1	0	3	13	
17	0	1	–	2	2	1	1	0		3	0	6	2	1	3	15	
18	0	1	–	2	2	1	1	0		3	0	6	2	1	3	15	
19	0	1	–	0	0	0	1	0		3	0	0	0	0	3	6	
20	0	1	–	1	2	1	1	0		3	0	3	2	1	3	12	

^a^Not available.

### Content Analysis

These sites were created by research and development organizations; research institutions; public institutions; and enterprises, such as those selling supplements, water, baby-related products, and insurance.

Each site was evaluated using content analysis to assess their “concerned terms of expression” and “accuracy of information.” Regarding concerned terms of expression, we identified inappropriate explanations concerning alcohol consumption and weight management during pregnancy and miscarriage. For example, while national policy recommends complete abstinence during pregnancy, some websites recommend reduced alcohol consumption without providing scientific evidence. Japanese guidelines advise weight gain based on prepregnancy BMI, but some sites recommended a uniform gain of approximately 11 kg, contradicting these standards. Discrepancies were found in the advised daily intake of iron. While some websites aligned with evidence-based guidelines from the Health Service Bureau, Ministry of Health, Labour and Welfare Japan [17], others cited outdated or inconsistent recommendations.

## Discussion

### Principal Results

We identified the top 20 websites presenting diet-related information for pregnant women using Google search and then assessed them by using QUEST. The results showed that, overall, the conflict of interest, and tone criteria scored higher, suggesting that the information is unbiased and is presented in a balanced way. Transparency was a concern, with 45% of websites lacking publication dates. Lower scores for complementarity and attribution indicated insufficient support for patient-physician relationships and gaps in scientific reliability [16].

In particular, all 20 sites surveyed scored 1 or higher for tone, suggesting that the website authors supported their claims with cautious statements but without discussing the claims’ potential limitations [16]. Notably, only 1 site had the highest tone score of 2. A maximum score of 2 indicates that the author’s claims are balanced by caution and state the potential limitations of their claims or contrasting evidence. Although all websites’ authors avoided absolute claims regarding the nutrition-related information they provided, pregnant women should be cautious about the possible limitations or potential contradictory claims of other information sources. This should be an essential criterion of information quality, especially when attribution scores are low.

Surprisingly, we found that Japanese websites presented scientifically inaccurate information regarding pregnancy nutrition, as indicated by the low attribution scores in the QUEST criteria. One-third of the sites provided no scientific evidence for their information, whereas the remaining were mainly based on expert opinions and unidentifiable research findings.

The analysis revealed significant inconsistencies in the guidelines cited for key topics. For example, advice on alcohol consumption ranged from abstinence to unsupported permissiveness. Similarly, weight management guidance often ignored prepregnancy BMI standards. Advice on nutrient intake, especially iron, was also inconsistent, with some sites citing outdated guidelines. The lack of trustworthiness of nutrition-related information for pregnancy might discourage information seekers from using that information [4]. Such reluctance of pregnant women to seek information from the internet with misleading sources might save them from misinformed dietary habits and health behaviors during pregnancy. Individuals with only basic education or an annual income below the national average have been found to possess inadequate health literacy [19]. Those with inadequate health literacy might use any information on the internet, resulting in adverse health outcomes for the mother and child because of their inability to evaluate conflicting nutritional information provided by various sources [20].

In an internet environment, where nutritional information is easily accessible, reliable evidence must be provided to protect mothers and children from any potential physical and psychological harm caused by misinformed behavior. Incorrect information without evidence may be displayed at the top of the search results and is likely to lead to its provision to users. Any harm caused by such incorrect dietary information during pregnancy must be avoided by any means possible. In addition, mothers tend to be concerned about and sensitive to nutrition during pregnancy not only because of their own physical changes but also because they are related to the growth of the fetus [21,22]. Pregnancy is inherently a stressful condition and pregnant women are particularly susceptible to psychological and physiological anxiety [21]. Prior research also indicates that pregnant women often experience confusion when confronted with conflicting health information [20]. As outlined by uncertainty theory [23], ambiguity, inadequacy, or inconsistency in health information can heighten anxiety and stress among patients, leading to emotional decision-making. Therefore, incorrect information, such as the findings of this study, may contribute to maternal anxiety. Policy makers and health care professionals should collaborate to establish quality standards, promote trusted platforms, and disseminate scientifically grounded information. In addition, efforts should focus on enhancing pregnant women’s health literacy to help them evaluate the reliability of information. In Japan, the government has introduced initiatives aimed at enhancing digital literacy and promoting fact-checking practices to verify the authenticity and timeliness of information [24]. However, public awareness of fact-checking remains relatively low compared to other developed countries [24]. This highlights the need to establish more effective systems for embedding such practices more broadly across society.

We need to highlight the low complementarity scores, which suggest a lack of support for the relationship between pregnant women and medical professionals. In particular, low complementarity scores of more than 70% of the sites suggest that, although the authors’ claims are implemented, subsequent engagement and relationship between the reader and professionals may need to be considered. Despite the accessibility benefits of internet-based nutritional information, verbal information provided by health care professionals remains the most important source of nutritional information during pregnancy [7]. Various studies have highlighted the importance of nutrition-related information that health care professionals, such as midwives and dietitians, provide through counseling with pregnant women [25-27].

### Limitations

This evaluation assessed the top 20 websites identified through Google Search using certain keywords and included only Japanese sites. This study could not assess the quality of all internet information, limiting the ability to generalize the results to Japan’s overall web landscape. Nevertheless, as the analyzed sites appear at the top of search results, they attract substantial visitor traffic. Evaluating the content quality of these highly ranked sites may aid future users and serve as a reminder to online information providers about the importance of accuracy. Future studies should incorporate additional search engines and refine search strategies to provide a more comprehensive view.

While QUEST is widely used for evaluating online health information, its application in the specific context of pregnancy nutrition may have limitations. Adjusting the importance of the criteria to align with the needs of specific user populations, such as pregnant women with complications, might be necessary. In addition, algorithmic bias in search engine rankings should be acknowledged. Despite efforts to reduce personalization, Google’s algorithm may reflect market trends or reinforce dominant perspectives, potentially limiting the diversity of visible content. While incognito mode helped minimize some personalization, other factors such as device fingerprinting or IP-based tracking may still have influenced the results. Future studies should consider using VPNs, anonymized environments, or multiple search engines to better account for these potential biases.

### Conclusion

The identified websites demonstrated careful vocabulary selection, avoiding an emphasis on claims that could lead to advertising or sales promotions. However, there was a notable lack of information supporting the patient-physician relationship, authorship details, and information update dates. Our findings underscore the need for improved standards in presenting online health information, particularly during pregnancy, to ensure accurate, reliable, and supportive content for pregnant women and health care providers.

Using web-based resources presents a convenient avenue for accessing desired information. Despite the presence of reputable and evidence-based sources, the prevalence of misleading information necessitates a critical approach. Hence, improving health literacy, especially regarding online information, is vital for readers to navigate and extract valuable insights from the multitude of online sources available. Future research should explore the role of digital health literacy in accessing reliable online health resources and promoting healthy behaviors during pregnancy.

## Abbreviations

QUEST: Quality Evaluation Scoring Tool
